# Effect of Preoperative Chronic Opioid Use on Mortality and Morbidity in Vascular Surgical Patients

**DOI:** 10.7759/cureus.20484

**Published:** 2021-12-17

**Authors:** Andras Szabo, Dominika Szabo, Krisztina Toth, Balazs Szecsi, Agnes Sandor, Rita Szentgroti, Boglar Parkanyi, Bela Merkely, Janos Gal, Andrea Szekely

**Affiliations:** 1 Department of Anesthesiology and Intensive Therapy, Semmelweis University, Budapest, HUN; 2 Heart and Vascular Centre, Semmelweis University, Budapest, HUN; 3 Schools of PhD Studies, Semmelweis University, Budapest, HUN

**Keywords:** risk management, preoperative assessment, vascular surgery, risk assessment tools, chronic opioid use, opioid epidemic

## Abstract

Introduction

Opioid derivates are an essential part of everyday clinical pain management practice. They have excellent analgesic, sedative, and sympatholytic effects and are widely used in various conditions. Beyond advantageous aspects, there are numerous problems with the chronic use of these agents. Dependency and life-threatening complications are the biggest problems with both illegal and prescribed opioid derivates. In our current study, effects of chronic opioid use were observed on mortality and life quality in the case of vascular surgery.

Methods

This prospective, observational study was conducted between 2014 and 2017. After obtaining informed consent, all participants were asked to fill a questionnaire containing different psychological tests. Perioperative data, chronic medical therapy, and anthropometric data were also collected. Opioid user and non-user patients’ psychological results were compared with non-parametrical tests. The effect of chronic opioid administration was investigated with logistic regression method with bootstrapping.

Results

Finally, the data of 164 patients were analyzed. 64.0% of participants were male, the mean age was 67.05 years, and the standard deviation was 9.48 years. The median follow-up time was 1312 days [interquartile range (IQR): 930-1582 days]. During the follow-up time, 42 patients died (25.6%). In the examined patient cohort, the frequency of opioid derivate use was 3.7% (only six patients). In the non-survived group, opioid use was significantly higher (1.6% vs. 9.5%, p=0.019). Significant differences were found in the aspect of cognitive performance measured by Mini-Mental State Examination (MMSE), opioid users have had lower points [25.5 (IQR: 24.5-26.0) vs. 28.0 (IQR: 27.0-29.0) p=0.008]. Opioid users have showed higher score on Beck Depression Inventory (BDI) [15.5 (IQR: 10.0-18.0) vs. 6.0 (IQR: 3.0-11.0), p=0.030). In a multivariate Cox regression model built up from registered preoperative medical treatment, opioids were found as a risk factor for all-cause mortality [adjusted hazard ratio (AHR): 4.31, 95% CI: 1.77-10.55, p=0.001].

Conclusion

Our current findings suggest that chronic, preoperative use of opioids could associate with increased mortality. Furthermore, both decrease in cognitive performance and increased depression symptoms were found in the opioid user cohorts which emphasize the importance of further risk stratification of these patients.

## Introduction

Opioid derivatives are an essential part of everyday clinical pain management practice. They have excellent analgesic, sedative, and sympatholytic effects and are widely used in various conditions.

According to its definition, opioids are generally synthetic or organic substances that act on opioid receptors (µ, κ, δ) and can be antagonized by naloxone. They are administered by different routes (intravenous, oral, transdermal, intrathecal, etc.) and their pharmacological parameters can vary widely.

In addition to the beneficial aspects, there are numerous problems associated with the chronic use of these drugs. The main disadvantages are the economic burden caused by substance addiction and the potential loss of life years due to opioid overdose [1,2]. Opioid abuse is a growing problem, affecting approximately 58 million people worldwide. The annual prevalence is highest in North America, Australia, and southwest Asia, reaching 2.5-3.5% per year, far above the global average (1.2% per year) across the population aged 15-64. In the USA in 2018, 3.7% of the population aged 12 or older (10.3 million people) misused opioid derivatives. According to the WHO drug report, nearly 50,000 people have died from opioid overdose in the USA in 2019. These deaths were attributed to opioid derivatives, mostly to fentanyl, which is a synthetic opioid. North America has seen a spike in opioid overdose deaths since the onset of the pandemic [3]. Apart from fatal events, opioid misuse is strongly associated with psychological, sociological, and criminal problems [4].

According to a review, the main risk factors of postoperative opioid use disorders are the following: history of (any kind of) substance abuse, any physical malady, mental health history, and use of sedative/hypnotic agents. However, the solution to the opioid problem is not the strongly restricted use of these potent analgetic agents, it would be rather the vigilance [5].

Objectives

In the present study, we focused on the long-term opioid usage among vascular surgery patients. The patient group with prescribed opioid derivatives often suffers from chronic pain, reduced quality of life, and obstructed mobility [6]. The use of opioids has been widely reported over the past decades in patients undergoing non-cardiac surgery [7,8]. The primary outcome was defined as the effect on overall mortality of chronic preoperative opioid usage. Relationship between preoperative opioid derivatives usage and operative risk estimated by vascular POSSUM (Physiological and Operative Severity Score for the enUmeration of Mortality and morbidity) score was investigated. Various psychological and cognitive test results were analyzed beyond negative outcomes caused by these drugs. Comprehensive frailty scores were also compared among patient groups.

Overcoming opioid-related addiction problems again requires major efforts and a multidisciplinary approach. Various combinations of minor and major analgesics or maybe other supplementary techniques are required, for example, neuromodulators such as antiepileptics and antidepressants [9].

## Materials and methods

Study design, setting, and participants

This prospective study was approved by the Regional Ethics Committee (TuKEB 250/2013) and registered on ClinicalTrials.gov (NCT02224222). In addition to the original aims/objectives, a sub-analysis has been carried out and is presented in this article. Study inclusion criteria were: age over 18 years, patients of elective vascular surgery. Patients were invited to participate in the study during their outpatient anesthesiology visit and were asked to complete baseline psychological questionnaires. After signing an informed consent, primarily 199 patients were included in the study who were admitted to the Department of Vascular Surgery at the Semmelweis University Centre for Cardiovascular Medicine, in Budapest, Hungary, between September 2014 and August 2017. Thirty two patients were excluded because of the cancellation of surgery and three patients because of withdrawal of their written consent. Thus, data of 164 patients was examined.

Definitions and measurements (variables, data sources, and grouping), bias, and study size

Basic anthropometric data, medical history, and prescribed drugs/medications were collected. Complete blood count, creatinine, serum urea nitrogen, and C-reactive protein levels were also measured. The completed questionnaires were structured to assess the psychological, functional, and cognitive status of the patients.

The Mini-Mental State Examination (MMSE) was used to map patients’ cognitive function. This questionnaire is widely used in everyday clinical practice and can identify considerable cognitive dysfunction in a very specific way [10]. It includes questions on psychological orientation, a short-term memory test, spelling tasks, and tests of sensory and motor function. The cut-offs for the 30-item scoring system were individually adjusted for age and educational level [11].

The Geriatric Depression Scale (GDS) is a special scoring system to measure major depression symptoms in the elderly. The 30-item self-report questionnaire contains “yes-or-no” questions. Scores between 10 and 19 indicate mild depression, while scores above 20 indicate severe depression [12].

The Beck Depression Inventory (BDI) was used to assess the severity of depression in vascular surgery patients. This is a 21-item, multiple-choice questionnaire. It has been used on the general population. Scores between 10 and 18 indicate mild, between 19 and 29 moderate, and scores above 30 indicate severe depression. Scores below 9 points suggest absence of depression symptoms [13].

The State-Trait Anxiety Inventory (STAI-T) was used to measure trait and state anxiety. The 20-item, 4-level Likert-type questionnaire has a cut-off point at 45. Below this value, the anxiety level of the patients is assumed probably normal [14].

The Caldwell Social Support Dimension Scale (CSSDS) was used to measure patients’ self-reported social support, including support received from (their) relatives and others [15]. Both the family and non-family axis were presented.

The Devins Illness Intrusiveness Rating Scale provided information on the health quality of the patients, measured by loss of activity and motivations in daily living according to specific diseases [16].

The Athens Insomnia Scale 5 is a shortened version of the original scale introduced in early 2000. It can be used as a basic test for sleeping disorders and disturbances. Its use is simple and effective, and it works well in everyday clinical practice. Scores above 4 may indicate the presence of sleeping disorders or insomnia [17].

Vascular POSSUM is a scoring system used to assess morbidity and mortality after surgery, in this special case vascular surgery. It depends on age, past medical history (cardiac and respiratory functions), present vital parameters and laboratory parameters (full blood count, renal function, ion balance), operation severity and complexity, estimated blood loss, presence of malignancy, and the mode of surgery (elective, urgent or emergency) [18,19].

In addition to the classic questionnaire items listed above, we used very simple self-report scales such as self-reported happiness, life-satisfaction, and health. Their impact on medical treatment and life expectancy is well documented [20,21].

By combining the medical history of cardiovascular (recurrent angina pectoris, atrial fibrillation or flutter, stroke or TIA, myocardial infarction or chronic ischemic heart disease, hypertension, diabetes mellitus) and other risk factors (anxiety, arthritis, chronic obstructive pulmonary disease, or asthma, cancer, chronic renal disease, degenerative spine disease), cognitive dysfunction, nutritional and functional status, a comprehensive frailty score was created. The frailty index was calculated as the frailty score divided by the number of predictors [22].

Long-term follow-up was carried out using the database of the National Health Insurance Fund of Hungary.

Outcomes

The primary outcome was the risk of overall postoperative mortality if chronic opioid use occurred during the follow-up period. In a further analysis, the interactions between chronic opioid use and cognitive dysfunction, depression and anxiety were described using MMSE and BDI, GDS and STAI-T scores, respectively.

Statistical analysis

Normality was tested using the Shapiro-Wilk test. Normal distributions are described by means and standard deviations, and non-normal distributions by medians and interquartile ranges 25-75 (IQR). Categorical data is presented as quantities and percentages and were evaluated using the Chi-Square test. Continuous variables were compared using the Mann-Whitney U test. Cox regression models were used to analyze mortality risk and Kaplan-Meier analysis to describe survival. Bootstrapping was used for crosstabulation and logistic regression methods [23]. The two-sided alpha level of 0.05 was applied.

Statistical analyses were performed using IBM-SPSS 24.0 software (International Business Machines Corporation, Armonk, New York, USA). Forest plot was created using GraphPad Prism version 8.0.0 for Windows (GraphPad Software, San Diego, California USA, www.graphpad.com”).

## Results

Descriptive data

Data of 164 patients was analyzed. 64.02% of the participants were male, with a mean age of 67.05 years and an SD of 9.48 years. The median follow-up time was 1312 days (IQR: 930-1582 days). During the follow-up period, 42 patients died (25.61%), 66.67% of non-survivors were male.

The surgeries were classified into four main groups: 1) surgeries on the descendent aorta, 2) the iliac system, 3) peripheral arteries, and 4) carotid arteries. Most of the interventions were performed on the carotid arteries (43.56%). The peripheral or extremities’ arterial system accounted for 20.24%, 14.11% on the iliac region, and 22.09% on the descending aorta. Applying crosstabulation analysis, neither primary nor secondary outcomes showed significant differences regarding the type of surgery.

Main results

The frequency of opioid derivative usage in the study group was 3.66% (6 patients). The patients used transdermal fentanyl or tramadol per os. The exact indication of opioid derivative usage was not recorded. In the group of non-survivors, opioid use was significantly higher (1.64% vs. 9.52%, p=0.019). The differences in demographic and preoperative medical treatments between survivors and non-survivors are shown on Table 1.

**Table 1:** Demographic data and preoperative medical treatments between survivor and non-survivor patient groups ACEI: angiotensin-converting enzyme inhibitor; ARB: angiotensin receptor blocker; BMI: body mass index; COPD: chronic obstructive pulmonary disease; IQR 25-27: interquartile range 25-75; OAC: oral anticoagulants; OAD: oral antidiabetics; PDE: phosphodiesterase; SSRI: selective serotonin reuptake inhibitor; TIA: transient ischemic attack; POSSUM: Physiological and Operative Severity Score for the enUmeration of Mortality and morbidity

		Survivors (n=122, 74.39%)				Non-survivors (n=42, 25.61%)				
		N	%	Median	IQR 25-75	N	%	Median	IQR 25-75	p-value
Gender	male	77	63.11%			28	66.67%			0.679
Age (years)				68.00	60.00-74.00			68.50	62.00-73.00	0.874
BMI				27.39	24.20-30.80			25.30	23.18-28.73	0.092
Vascular POSSUM				16.00	14.00-18.00			17.00	15.00-22.00	0.030
Ischemic heart disease		43	35.25%			15	35.71%			0.956
Diabetes mellitus		35	28.69%			19	45.24%			0.049
Hypertension		108	88.52%			34	80.95%			0.214
Obesity (BMI≥30)		31	25.41%			5	11.90%			0.068
Neoplasia		28	22.95%			10	23.81%			0.909
Psychiatric disorder		5	4.10%			3	7.14%			0.430
Previous vascular surgery		53	43.44%			28	66.67%			0.009
Stroke or TIA		20	16.39%			11	26.19%			0.162
COPD		25	20.49%			14	33.33%			0.092
Acetylsalicylic acid		70	57.38%			26	61.90%			0.607
Clopidogrel		32	26.23%			6	14.29%			0.114
Apixaban		3	2.46%			0	0.00%			0.305
Other antiplatelet drugs		2	1.64%			0	0.00%			0.404
OAC		5	4.10%			2	4.76%			0.854
PDE inhibitor		9	7.38%			3	7.32%			0.99
Benzodiazepine		34	27.87%			9	21.43%			0.413
SSRI		7	5.74%			2	4.76%			0.811
Other antidepressants		4	3.28%			0	0.00%			0.235
Beta-blockers		63	51.64%			15	35.71%			0.075
Calcium channel blockers		46	37.70%			13	30.95%			0.432
ACEI		55	45.08%			21	50.00%			0.581
ARB		17	13.93%			6	14.29%			0.955
Diuretics		54	44.26%			25	59.52%			0.088
Digitalis		3	2.46%			3	7.14%			0.163
OAD		20	16.39%			12	28.57%			0.086
Insulin		7	5.74%			5	11.90%			0.186
Antiepileptics		3	2.46%			1	2.38%			0.977
Steroid		9	7.38%			4	9.52%			0.657
Statin		68	55.74%			19	45.24%			0.240
Opioid derivatives		2	1.64%			4	9.52%			0.019

Regarding overall mortality, the use of opioid derivatives appeared to be an independent risk factor according to the univariate Cox regression model [hazard ratio (HR): 2.49, 95% CI: 1.20-5.18, p=0.014] and V-POSSUM score-adjusted Cox regression model [adjusted hazard ratio (AHR): 2.40, 95% CI: 1.15-5.01, p=0.020].

**Figure 1:**
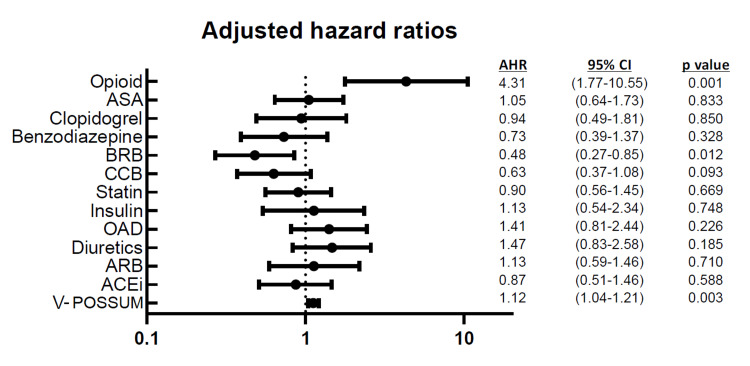
Multivariate Cox regression model with different factors for all-cause mortality ASA: acetylsalicylic acid; ACEi: angiotensin-converting enzyme inhibitor; AHR: adjusted hazard ratio; CI: confidence interval; ARB: angiotensin receptor blocker; BRB: beta-receptor blocker; CCB: calcium channel blocker; OAD: oral antidiabetics; V-POSSUM: Vascular Physiological and Operative Severity Score for the enUmeration of Mortality and morbidity

In a multivariate Cox regression model, opioids were found to be an independent risk factor for all-cause mortality (AHR: 4.31, 95% CI: 1.77-10.55, p=0.001). The use of beta-blockers was found to have a protective effect (AHR: 0.48, 95% CI: 0.27-0.85, p=0.012). Vascular POSSUM score was a significant predictor of overall (HR: 1.12, 95% CI: 1.04-1.21, p=0.003). The entire model is represented as a forest plot in Figure 1.

Relationship between psychological variables and opioid use

Various psychological parameters were analyzed regarding opioid usage. Significant differences were found regarding cognitive function measured by the MMSE, with opioid users scoring lower [25.50 (IQR: 24.50-26.00) vs. 28.00 (IQR: 27.00-29.09, p=0.008]. Opioid users reached higher scores on the BDI [15.50 (IQR: 10.00-18.00) vs. 6.00 (IQR: 3.00-11.00), p=0.030]. The results of all completed inventories are shown in Table 2.

**Table 2:** Results on different psychological inventories and on Vascular POSSUM according to use of opioid derivatives GDS: Geriatric Depression Scale; IQR 25-75: interquartile range 25-75; LOS: length of stay; MMSE: Mini-Mental State Examination; STAI-T: State-Trait Anxiety Inventory—T axis; POSSUM: Physiological and Operative Severity Score for the enUmeration of Mortality and morbidity

	Use of opioid derivates						
	No			Yes			
	Median	IQR 25-75		Median	IQR 25-75		p-value
MMSE Score	28.00	27.00	29.00	25.50	24.00	26.00	0.008
GDS Score	5.00	2.00	7.00	5.50	5.00	7.00	0.626
BDI Score	6.00	3.00	11.00	15.50	10.00	18.00	0.030
STAI-T Score	40.50	35.00	51.00	42.50	29.00	51.00	0.830
Self-rated satisfaction (1-10)	7.00	5.00	8.00	5.50	1.00	6.00	0.118
Self-rated happiness (1-10)	7.00	5.00	9.00	5.00	4.00	6.00	0.036
Athens Insomnia Scale 5	1.00	0.00	3.00	2.00	0.00	4.00	0.462
Comprehensive Frailty Score	4.00	3.00	6.00	6.80	5.30	8.00	0.018
In-hospital LOS (days)	7.00	5.00	10.00	12.00	7.00	15.00	0.120
Ward LOS (days)	6.00	5.00	9.00	12.00	7.00	13.00	0.062
Vascular POSSUM	16.00	14.00	19.00	15.00	13.00	24.00	0.689

In the opioid user group, self-rated happiness scores were found to be lower [7.00 (IQR: 5.00-9.00) vs. 5.00 (IQR: 4.00-6.00), p=0.036] and self-rated life-satisfaction was also lower in a non-significant way [7.00 (IQR: 5.00-8.00) vs. 5.50 (IQR: 1.00-6.00), p=0.116].

The calculated comprehensive frailty score increased among patients who were chronic users of opioid derivatives [4.00 (IQR: 3.00-6.00) vs. 6.80 (IQR: 5.80-8.00), p=0.018].

A considerable tendency was observed in-hospital length of stay (LOS) [6 days (IQR: 5-9) vs. 12 (IQR: 7-13), p=0.068]. There was also a significant difference in hospital LOS according to vascular POSSUM score [16.00 (IQR: 14.00-18.00) vs. 17.00 (IQR: 15.00-23.00), p=0.030].

## Discussion

Our current findings show that chronic opioid use before surgery may be an independent risk factor for mortality after vascular surgery. Furthermore, chronic opioid derivative usage was associated with more frequent symptoms of depression measured by BDI and decreased cognitive function measured by MMSE. The comprehensive frailty score was also increased among patients with chronic opioid use. Our observational, prospective clinical study was conducted in a fairly homologous vascular surgical group. A long-term follow-up was performed to identify late complications and mortality.

As reported in the literature review earlier, opioid and opioid derivative use is associated with higher morbidity and mortality after colorectal surgery (HR for morbidity: 1.43, 95%CI 1.07-1.91, p<0.05 and HR for mortality: 1.48 95% CI: 1.05-2.08, respectively) [24]. In our dataset, we found similar results, with a significantly higher risk of mortality among preoperative opioid users (AHR: 4.31, 95% CI: 1.77-10.55, p=0.001). Aizpuru et al. described longer hospital stays for vascular surgery patients using preoperative opioids, but no significant difference in postoperative mortality [25]. The remarkable difference regarding our dataset is the increased risk of mortality. Similarly, a longer in-hospital stay was also described. Although readmission was not recorded and analyzed in our current study, the negative impact of pharmaceutical substance use following cardiac surgery has been described previously in a previous study [26].

Consistent with the results presented, previous studies have shown that opioid users have a higher risk of cognitive impairment [27]. We found significantly modest performance on the MMSE in patients with chronic preoperative opioid usage.

Previous observations showed that opioid use has an impact on depression severity and health-related quality of life but no clear relationship was demonstrated on the GDS score, nor on the anxiety axis as measured by STAI-T [28]. We measured the impact of chronic opioid use on depression’s symptoms on GDS and BDI scores. Consistently with earlier studies, our results described significantly higher BDI score among opioid users.

Vascular POSSUM is a widely used risk stratification system that can provide a relatively accurate estimation of the risk of postoperative mortality and morbidity [29,30]. By using the frailty score, the estimation became more accurate, the prediction of long-term mortality was significant. Our current results suggest that the simultaneous use of several risk estimation scales and different indicators may help to predict mortality and psychological morbidity in the short and long term.

Limitations of the study

The single-center study design and relatively small sample size, especially the low number of opioid users, did not allow for sufficient statistical power and strict/rigorous power of the adjustment. Further investigation is needed to distinguish between these psychological variables, which have a rather large variance.

Future studies and prospects

Current meta-analysis suggests that there is no strong evidence for the efficacy or safety of opioid weaning programs prescribed for chronic pain. Only a few randomized controlled trials have investigated psychological, pharmacological, and other types of interventions to reduce opioid use in patients with chronic pain [31]. However, interesting studies done were of pain and anxiety management with music therapy summarized in a recently published meta-analysis. After the clear benefit on reported pain and anxiety, a strong evidence of an advantageous effect on opioid usage was not confirmed [32]. Consequently, further randomized controlled trials regarding effective intervention methods are needed.

## Conclusions

Among an endless number of risk stratification scoring systems, clinicians must find the ones helping them effectively predict the potential risk factors for mortality and postoperative morbidity. Our current study suggests that chronic, preoperative opioid use has been associated with increased postoperative mortality. The increasing number of patients who regularly use opioid derivatives (either medicinally or illicitly) further emphasizes the importance of the current findings. In accordance to previous findings in the literature increased mortality, an impact on the incidence and severity of depression symptoms and cognitive impairment was also demonstrated.
